# Decreased Dp71 expression is associated with gastric adenocarcinoma prognosis

**DOI:** 10.18632/oncotarget.10724

**Published:** 2016-07-20

**Authors:** Sipin Tan, Jin Tan, Sichuang Tan, Shuai Zhao, Xiaoxia Cao, Zhikang Chen, Qiaocheng Weng, Huali Zhang, Kang kai Wang, Jiang Zhou, Xianzhong Xiao

**Affiliations:** ^1^ Laboratory of Shock, Department of Pathophysiology, Xiangya School of Medicine, Central South University, Changsha, Hunan 410008, People's Republic of China; ^2^ Department of Thoracic Surgery, Second Xiangya Hospital, Central South University, Hunan 410011, People's Republic of China; ^3^ Department of General Surgery, Xiangya Hospital, Central South University, Changsha 410008, People's Republic of China

**Keywords:** Dp71, lamin B1, gastric cancer, gastric cancer cells, prognosis

## Abstract

For the first time, dramatically decreased Dp71 protein and mRNA was found in 34 pairs of resected primary gastric adenocarcinoma. Immunohistochemistry identified Dp71 expression suppressed in 72.2% of 104 gastric cancer patients. The decreased Dp71 expression was significantly correlated with cancer differentiation (P=0.001) and lymph vascular invasion (p=0.041). Decreased Dp71 expression was associated with a poor gastric adenocarcinoma prognosis (P=0.001). Significantly less Dp71 mRNA and protein were found in BGC823, SGC7901, AGS compared with GES-1. Via increasing lamin B1 mRNA and protein, enforced Dp71d and Dp71f expression resulted in SGC7901 proliferation inhibition. Co-IP proved interaction of Dp71 with lamin B1 in GES-1 cells. Further expression characterization showed reduced lamin B1 in gastric cancer tissue and cancer cells. Increasing lamin B1 expression results in the growth inhibition of SGC7901, which suggests that Dp71-lamin B1 protein complex plays an important role for the newly identified tumor suppressive function of Dp71.

## INTRODUCTION

Cancer is a broad group of diseases in which cells divide and grow uncontrollably. Gastric cancer remains the fourth most common cancer in the world and the second most common cause of cancer-related deaths [1]. In China, gastric cancer was predicted to rank as the third most common cancer in 2005, with 0.3 million deaths and 0.4 million new cases reported [2, 3]. Although the incidence of gastric cancer has decreased over the past few decades, it is still necessary to identify new suitable markers to treat and predict the prognosis of gastric cancer.

Dp71 is one of the most widely distributed isoforms of dystrophin, the pathogenic gene of Duchenne muscular dystrophy [4]. Alternative spicing of dystrophin produced two major Dp71 isoforms: Dp71d and Dp71f. Dp71d was found to be a nuclear isoform, while Dp71f is the dominant cytoplasmic one [5-7]. Current Dp71 researches identified its involvement in ion and water homeostasis, cell signaling, cell adhesion, and nuclear architecture. Identified to be a component of the mitotic spindle and cytokinesis multi-protein apparatuses, Dp71 was also found to modulate the cell division [8-10].

Cancer is a type of disease involving uncontrollable cell proliferation. Because Dp71 is a component of the mitotic spindle, the first Dp71 mRNA and protein expression analysis in gastric cancer and cancer cell lines was made in our laboratory. Furthermore, the relationship between Dp71 protein and the clinicopathological features of gastric cancer and its prognostic value were also evaluated. Proliferation of SGC7901 was measured after the Dp71 expression was increased. Our further steps to characterize lamin B1 and lamin B1-Dp71 protein complex in gastric cancer tissues and cell lines proved that lamin B1 to be the putative target molecule for the newly identified tumor suppressive role of Dp71.

## RESULTS

### Dp71 expression was suppressed in gastric cancer

**Figure 1:**
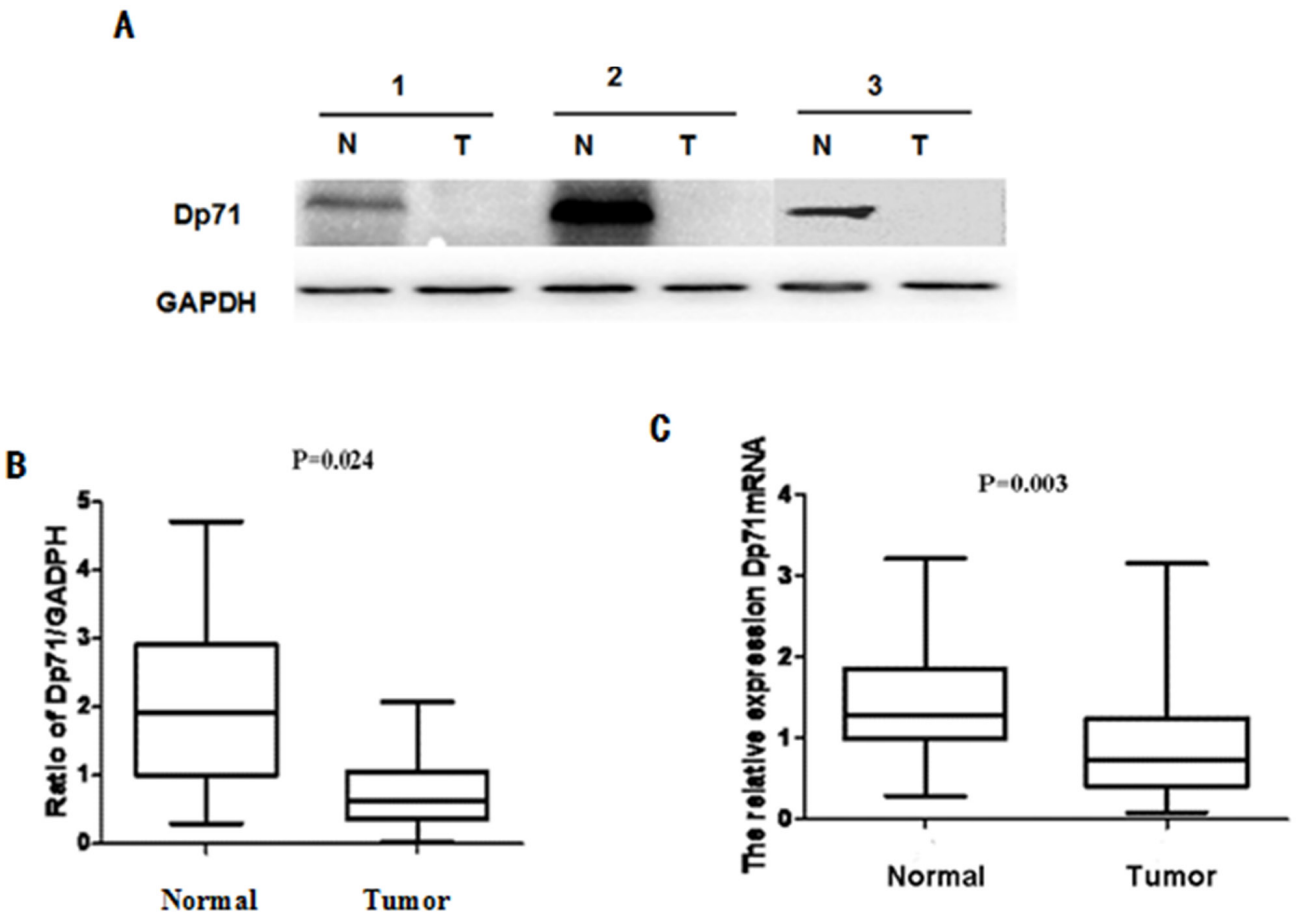
Dp71 expression decreased significantly in gastric cancer **A.** Dp71 protein expression in 34 paired gastric tissues assessed by western blotting. **B.** Statistical analysis of Dp71 protein expression (P = 0.024). **C.** Dp71 mRNA expression in 34 paired gastric tissues assessed by qRT-PCR (P=0.003). Real time RT-PCR was performed to detect Dp71 RNA expression in gastric cancer and adjacent non-cancer tissues, statistically less Dp71 mRNA were expressed in gastric cancer tissues.

The critical structural role of Dp71 in mitosis prompted us to determine whether this gene has a role in gastric cancer. The Dp71 protein levels in the 34 pairs of resected specimens (tumor tissue samples and matched adjacent non-tumor tissue samples) from gastric cancer patients were evaluated by western blot analysis. The results showed a Dp71 band at 71 kDa in matched non-tumor tissues while its expression decreased in tumor tissues. A decrease in Dp71 expression was observed in 27 pairs (71.8%) of the gastric tumor tissues analyzed (Figure 1A).

To determine whether this decreased expression occurred at the transcriptional level, Dp71 mRNA expression was determined with qRT-PCR assays using the same 34 pairs of resected specimens. The Dp71 mRNA levels were significantly reduced in 26 (69.2%) tumor tissue samples, compared with its levels in the adjacent non-tumor tissue samples (P = 0.003, Figure 1B). These results show that Dp71 expression was down-regulated in gastric cancer not only at the protein level but also at the RNA level.

### Down-regulated Dp71 protein expression was associated with gastric cancer differentiation and poor prognosis

Immunohistochemistry analyses were made in 104 paraffin-embedded gastric cancer sections. The positive expression of Dp71 was localized in both the nucleus and cytoplasm. Among the 104 gastric cancer sections, 29 cases (27.8%) showed high Dp71expression (Dp71 ++ or Dp71 +++), whereas the remaining 75 cases (72.2%) displayed low Dp71 expression (Dp71- or Dp71 +).

**Figure 2:**
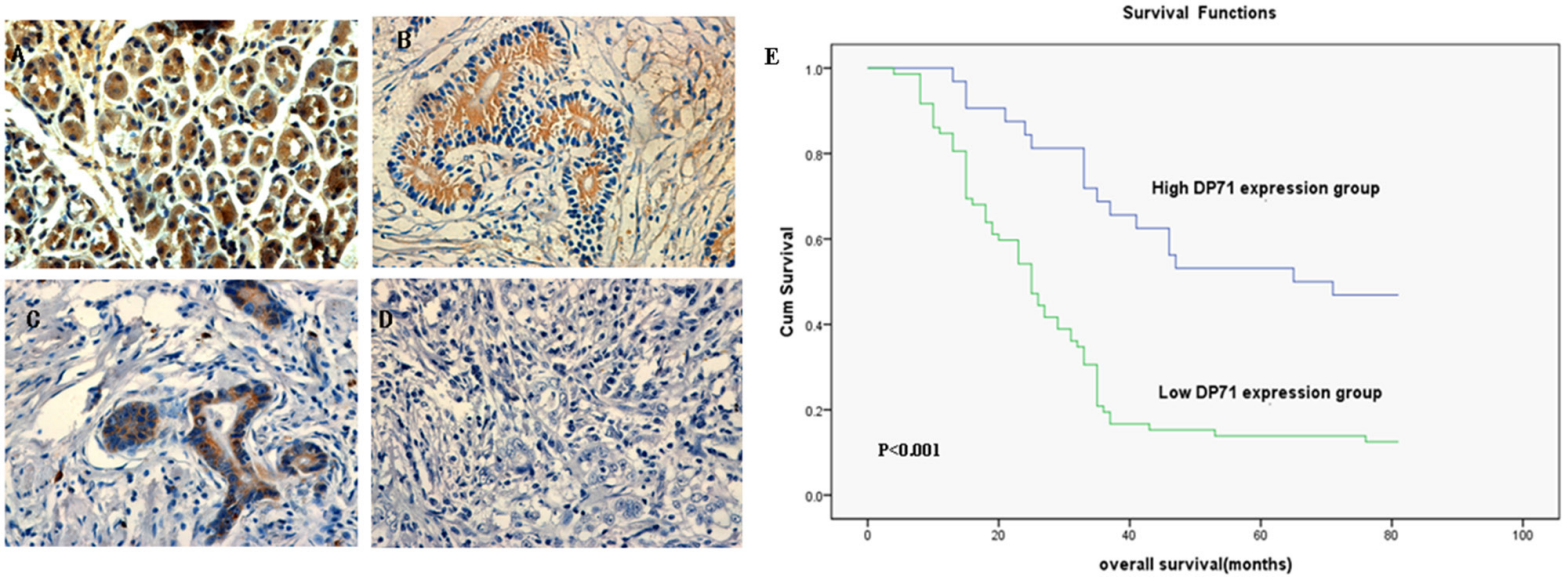
Immunohistochemistry of Dp71 expressions in gastric cancer and its prognostic implications **A.** Normal gastric tissues, scored as Dp71 (+++); **B.** Well-differentiated gastric cancer, scored as Dp71 (++) ; **C.** Moderately differentiated gastric cancer, scored as Dp71 (+); **D.** Poorly differentiated gastric cancer, scored as Dp71 (-). Original magnification: A–D ×400. **E.** Kaplan–Meier survival curves of gastric cancer patients (n = 104) after surgical resection. Decreased Dp71 expression correlated with poor patient survival. Patients in high Dp71 expression group exhibited significantly better survival than the low Dp71 expression group (log-rank test: P<0.001).

Normal gastric tissues showed the strongest Dp71 positive staining (Figure 2A). Well-differentiated cases showed strongly positive Dp71 expression (Dp71 ++) (Figure 2B), moderately-differentiated cases showed weakly positive expression (Dp71+) (Figure 2C), and the most poorly differentiated cases used showed no detectable Dp71expression (Dp71-) (Figure 2D).

Based on the categories that we defined in the previously mentioned methods, the data showed that there is no apparent association between Dp71 expression and clinical stages, patients’ ages, tumor invasion, or tumor size. The low Dp71 expression was significantly correlated with cancer differentiation (r = 0.171, P= 0.001), and lymph vascular invasion (r = 0.123, P= 0.041) (Table 1).

**Table 1:** Correlations between Dp71 expression and clinic pathologic variables in 104 gastric cancer patients

Variables	Number(%)	DP71 expression		P
		High(%)	LOW(%)	
Gender				0.140
Male	72	20	52	
Female	32	9	23	
Age(years)				0.740
< 60	72	22	50	
≥60	32	7	25	
Differentiation				0.000**
High	18	14	4	
Low	86	15	71	
Tumor invasion				0.760
T1-T2	39	19	20	
T3-T4	65	10	55	
TNM stage				0.552
I~II	30	16	14	
III~IV	74	13	61	
Lymph vascular invasion				0.008**
No	58	23	35	
Yes	46	6	40	
Tumor size				0.878
< 5cm	35	17	18	
≥ 5cm	69	12	57	

The median survival time of the 104 gastric cancer patients was 38 months(range 7-98 months). The overall survival rate in the high Dp71 expression group was significantly improved compared to the low expression group [56.56% vs. 30.8% (5-year survival rate), P < 0.001, Figure 2E].

### Decreased Dp71 mRNA and protein expression in gastric cancer cell lines

**Figure 3:**
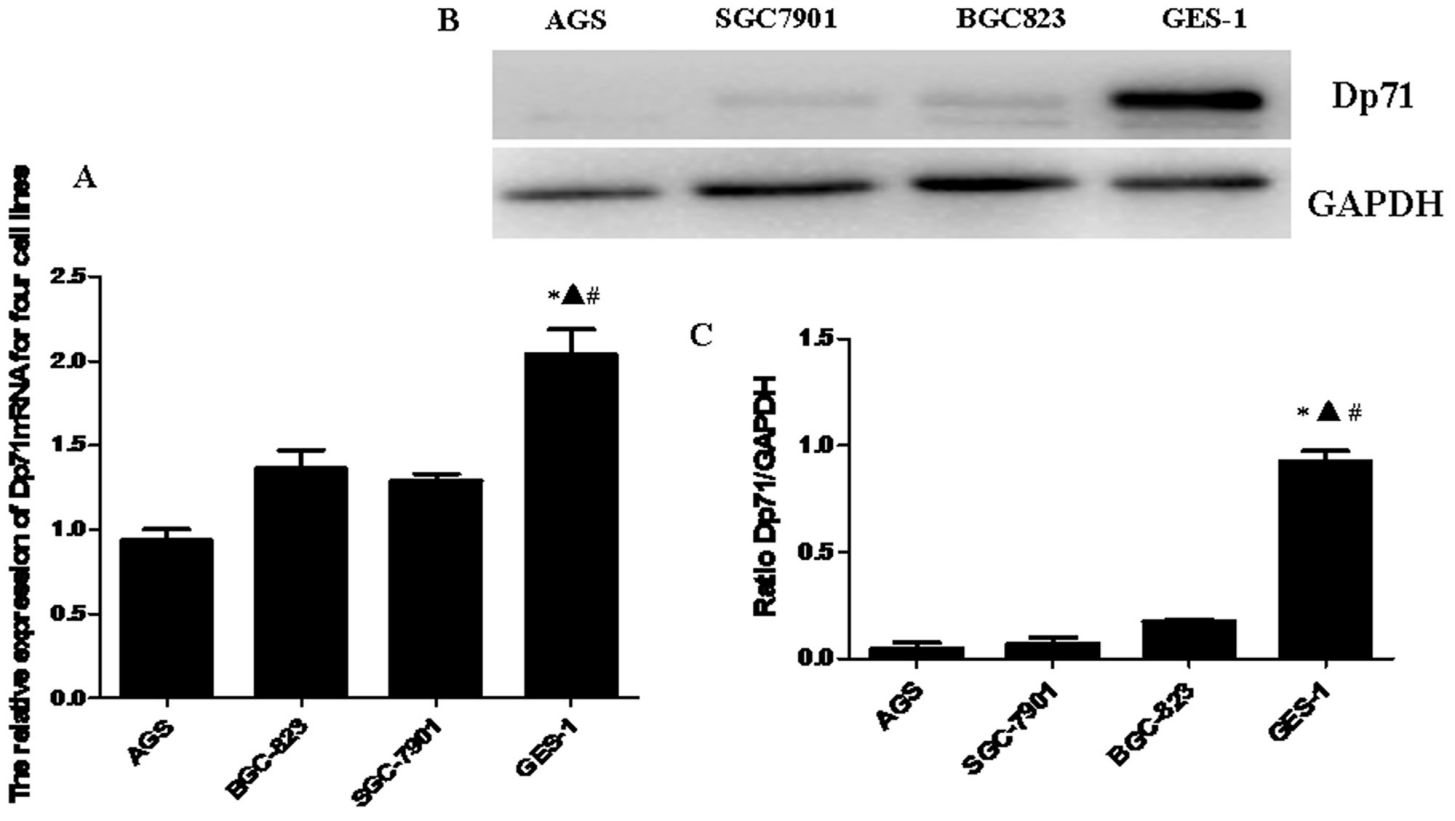
Decreased Dp71 mRNA and protein expression in gastric cancer cell lines **A.** RT-qPCR analysis revealed that the Dp71 mRNA expression was down-regulated in the AGS, BGC823, and SGC7901 cells compared with the normal gastric epithelial cell line GES1. *P <0.05, GES-1 versus AGS, ▲ *P < 0.05*, GES-1 versus BGC-823, #*P < 0.05*, GES-1 versus SGC-7901. **B.** Western blotting analysis of Dp71 expression in GES-1 and and AGS, BGC823, and SGC7901 cells. **C.** Statistical analysis of Dp71 protein expression of GES-1 and AGS, BGC823, and SGC7901 cells. *P < 0.05, GES-1 versus AGS, ▲ *P < 0.05*, GES-1 versus BGC823, # *P < 0.05*, GES-1 versus SGC-7901. There are no statistical differences between AGS, BGC823, and SGC7901 cells.

Real-time quantitative PCR was performed to determine the Dp71 mRNA levels of normal gastric epithelial cell line GES-1 and gastric cancer cell lines AGS, SGC7901 and BGC823. Dp71 expression level was significantly lower in the gastric cancer cell lines than in the GES-1 normal gastric epithelial cell line (Figure 3A).

Western blot analysis was further made in GES-1 and three gastric cancer cell lines to analyze the Dp71 expression. Consistent with the qRT-PCR results, as indicated in Figure 3B, significantly less protein expression was observed in the three gastric cancer cell lines compared with normal human gastric epithelial cell line GES-1, while there were no significant statistical differences in Dp71 protein expression among the three cancer cell lines (Figure 3C).

### SGC7901 cells overexpression Dp71 displayed proliferation inhibition

**Figure 4:**
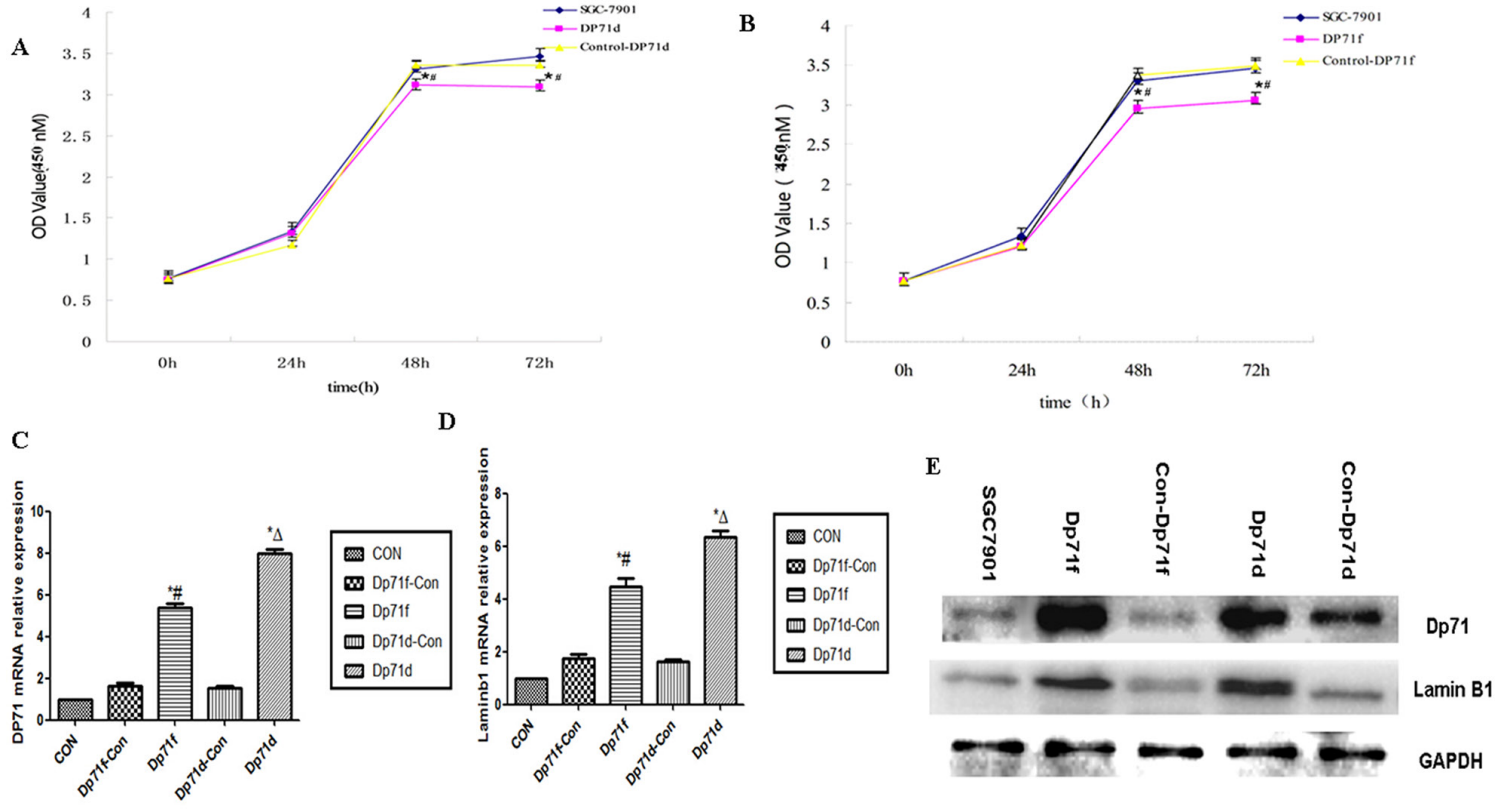
Increasing Dp71 expressions in SGC7901 cell inhibited its proliferation **A.** SGC7901-Dp71d cells displayed inhibited proliferation, *P<0.05, versus SGC7901-Control Dp71d cells; #*P<0.05*, versus blank SGC7901 cells. **B.** SGC7901-Dp71f cells displayed inhibited proliferation; *P < 0.05, versus SGC7901-Control Dp71f cells; #*P < 0.05,* versus blank SGC7901 cells,. **C.** SGC7901-Dp71d and SGC7901-Dp71f cells increased their Dp71 mRNA and protein **D.** SGC7901-Dp71d and SGC7901-Dp71f cells increased their laminB1 mRNA expression. SGC7901-Dp71d and SGC7901-Dp71f cells increased their laminB1 mRNA expression compared with SGC7901-Control Dp71d, SGC7901-Control Dp71f and parental cells. *P < 0.05, versus blank SGC7901 cells; Δ *P <0.05*, versus SGC7901-Control Dp71d cells; #*P < 0.05*, versus SGC7901-Control Dp71f cells. **E.** Increased lamin B1 and Dp71 protein in SGC7901-Dp71d and SGC7901-Dp71f cells. After transfection of Dp71d and Dp71f plasmids in SGC7901 cells, western blot were performed to detect Dp71 and lamin B1 expression in SGC7901, SGC7901-Dp71d, SGC7901-Dp71f, SGC7901-Control Dp71d, SGC7901-Control Dp71f cells, lamin B1 and Dp71 protein expression were significantly increased in SGC7901-Dp71d and SGC7901-Dp71f cells.

CCK8 assay was then performed to detect proliferation rate of both SGC7901-Dp71d and SGC 7901-Dp71f cells. As indicated in Figure 4A and Figure 4B, significant growth inhibition can be observed 48h, 72h after the Dp71f and DP71d plasmids were transfected into SGC7901 cells, while there was no statistical difference between the control vector group and the blank cell group. After the Dp71f and Dp71d plasmids were transfected into the SGC7901 cells for 48 hours, Q-RT-PCR displayed increased Dp71, lamin B1 mRNA expression in SGC7901-Dp71d and SGC 7901-Dp71f cells. Western blot analyses showed fold increase of Dp71 and lamin B1 protein. As indicated in Figure 4C and 4E, lamin B1 mRNA and protein expression were both increased in SGC7901-Dp71d and SGC7901-Dp71f cells, which is consistent with previous research on Dp71 and lamin B1 expression in PC12 cells.

### Interaction between Dp71 and lamin B1 in GES-1 cells

**Figure 5:**
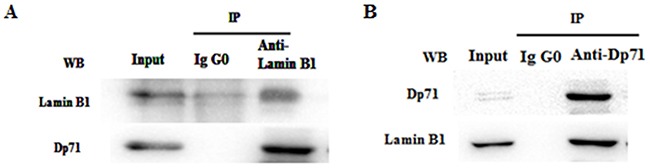
Dp71 interacts with lamin B1 in GES-1 cells Whole cell extracts from GES-1 cells were immunoprecipitated with a rabbit anti-lamin B1 antibody **A.** rabbit anti-Dp71 antibody **B.** an irrelevant antibody (IgG0) was used as control. Immunoprecipitated proteins were analyzed by western blotting with mouse anti-lamin B1 and anti-Dp71 antibodies. Association between Dp71 and lamin B1 was observed in GES-1 cells.

In order to prove interaction between Dp71 and lamin B1 in gastric epithelium cells, total protein extracts from GES-1 cells were immunoprecipitated with an anti-lamin B1 or anti–Dp71 antibody, precipitated proteins were analyzed by immunoblotting with antibodies directed specifically to either lamin B1 or Dp71. Figure 5A showed that Dp71 was pulled down together with lamin B1 by the anti-lamin B1 antibody in GES-1 cells, whereas none of these two proteins was recovered when an irrelevant antibody (IgG0) was used for immunoprecipitation, thus establishing the specificity of the assays. In Figure 5B, lamin B1 was pulled down together with rabbit anti-Dp71 antibody. Co-immunoprecipitation with each specific antibody proved association between Dp71 and lamin B1 in GES-1 cells, which proved the formation of lamin B1-Dp71 protein complex in GES-1 cells.

### Decreased lamin B1 in gastric cancer tissues and gastric cancer cells

**Figure 6:**
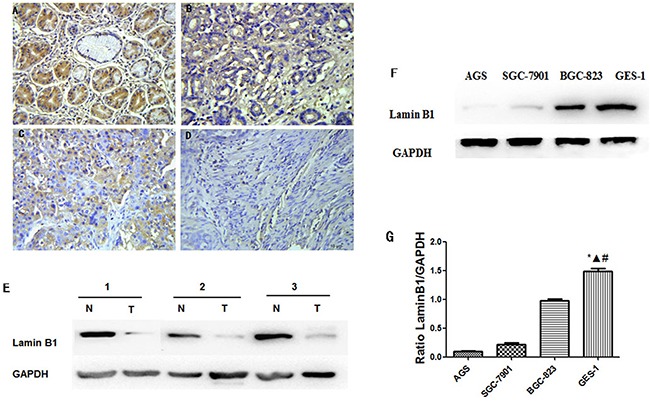
Decreased lamin B1 expressions in gastric cancer tissue and cancer cells Normal gastric tissues, scored as lamin B1 (+++); **B.** Well-differentiated gastric cancer, scored as lamin B1 (++) ; **C.** Moderately differentiated gastric cancer, scored as lamin B1 (+); **D.** Poorly differentiated gastric cancer, scored as lamin B1 (-). Original magnification: A–D ×400. **E.** Western blot analysis identified reduced lamin B1 protein in gastric cancer tissues. **F.** Lamin B1 protein decreased in gastric cancer cell lines, western blot analysis identified reduced lamin B1 protein expression in AGS, BGC823, and SGC7901 cells compared with GES-1 cells. **G.** Statistical analysis of lamin B1 protein expression in GES-1, AGS, BGC823, and SGC7901 cells. Statistically less lamin B1 protein was expressed in gastric cancer cells compared with GES-1 cell. *P < 0.05, GES-1 versus AGS, Δ*P < 0.05*, GES-1 versus BGC823, # *P < 0.05*, GES-1 versus SGC-7901.

Lamin B1 is an important nuclear cytoskeleton protein interacting with Dp71. In our further exploration of lamin B1 in gastric cancer, immunohistochemistry analysis was carried out in 75 paraffin-embedded gastric cancer sections; the results were consistent with previous lamin B1 characterization in gastric cancer [20]. Normal gastric tissues showed the strongest lamin B1 positive staining, well-differentiated cases showed strongly positive lamin B1 expression, moderately-differentiated cases showed weakly positive expression, and the most poorly differentiated cancer samples show no detectable lamin B1 expression (Figure 6A-6D). Western blot analysis of fresh resected primary gastric adenocarcinoma tissues displayed reduced lamin B1 expression, as indicated in Figure 6E. In gastric cancer cell lines AGS, SGC7901 and BGC823, decreased lamin B1 expression was also identified compared with GES-1 cells. The differences between GES-1 and the gastric cancer cells were statistically significant. In summary, the expression profiles of lamin B1 are consistent with Dp71 in gastric cancer tissues and cancer cells.

### Proliferation inhibition of lamin B1 over expression SGC7901 cells

**Figure 7:**
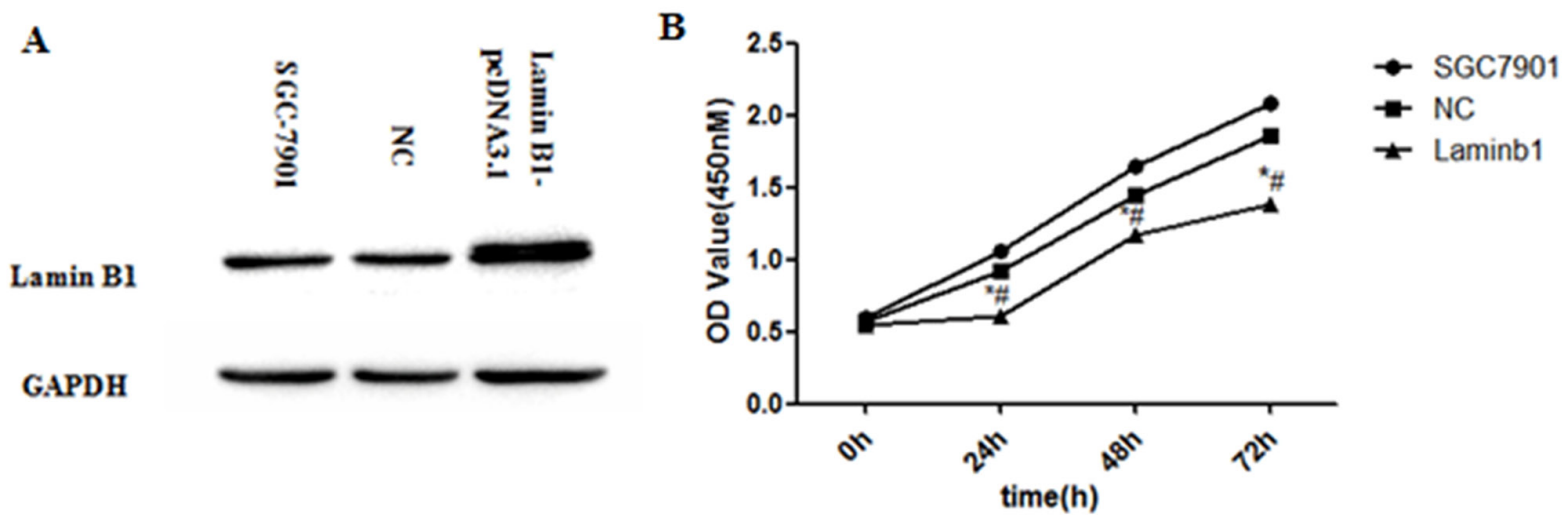
Increased lamin B1 expression inhibited the proliferation of SGC7901 cells **A.** Increased lamin B1 protein was detected in SGC7901 cells after transfection of lamin B1-pcDNA3.1 plasmids **B.** Increasing lamin B1 in SGC7901 displayed inhibited proliferation; *P < 0.05, SGC7901-lamin B1 cells versus SGC7901-Control, # *P < 0.05*, SGC7901-lamin B1 cells versus blank SGC7901 cells.

Lamin B1-pcDNA3.1 plasmid was constructed and transfected into SGC7901 cells to verify its tumor suppression function. Significant growth inhibition of SGC7901 cells can be observed 24h, 48h, and 72h after lamin B1 plasmids were transfected (Figure 7A and 7B). The growth alteration of SGC7901-lamin B1 cell line showed that increasing lamin B1 expression can inhibit its proliferation, which presented the evidence of a tumor suppressive role of lamin B1 in gastric cancer.

## DISCUSSION

Being the most ubiquitous expressed isoform of dystrophin, Dp71 was found to play various functions in cell signaling, adhesion, cell cycle division, and nuclear architecture [8-10, 13, 14]. Because mitotic spindle abnormality is a common cause for cancer [15, 16], its indispensable role in mitotic spindle sets us starting our research on Dp71 expression in gastric adenocarcinoma. For the first time, decreased Dp71 mRNA and protein in gastric cancer tissues and gastric cell lines was displayed. It is also showed that reduced Dp71 expression was associated with cancer differentiation and lymph vascular invasion. Up-regulating Dp71 expression in SGC7901 cells results in significant growth inhibition. Clinical and cellular experiments highly suggest a tumor suppressive role Dp71 played in gastric cancer.

How did the decreased Dp71 expression occur in gastric cancer? The cellular content of Dp71 protein and mRNA varies in different cells via transcriptional regulation of different proteins [17, 18]. Bioinformatics analysis identified two highly possible binding sites for HNF3α (-1596 ~ -1611, -1935 ~ -1950) and KLF4 (-190 ~ -200,-711 ~ -721) in the promoter region of Dp71. KLF4 protein was significantly decreased in gastric cancer samples compared with normal gastric mucosa [19, 20]. Our preliminary immunohistochemistry work identified reduced expression of HNF3α in gastric cancer (unpublished data). Increasing KLF4, HNF3α in SGC7901 cells caused Dp71 protein fold increase. Knocking KLF4, HNF3α down caused expression decrease of Dp71 in GES-1 cells, which suggests that both proteins act as a positive regulator of Dp71 (See supplementary results). Functioning as one of the down stream target proteins of these putative tumor suppressive genes, the reduced expression of Dp71 might serve as an important molecule events leading to the further change of gastric epithelium. Obvious growth inhibition of SCG7901-Dp71f and SCG7901-Dp71d cells were displayed in our work, which is consistent with the phenotypes of N87 and SK-GT5 gastric cancer cell lines after the restoration of KLF4 expression [21].

It is interesting that enhanced Dp71d and Dp71f expression in SGC7901 cells showed the same phenotypes, for the Dp71d is proposed to be nuclear protein while Dp71f is mainly cytoplasmic. However, current research displayed that the location of Dp71f and Dp71d varies in different cells. Research showed that ZZ finger motifs of Dp71, which encoded by exons 68 and 69, are the main nucleus translocation sequence. In HEK293 cells, Dp71f family members displayed a dominant nucleus location. In cultured bipolar GABAergic and multipolar Glutamatergic neurons, both Dp71d and Dp71f were shown to form a nuclear DAPC complex. Cytoplasmic and nuclear shuttling movements of Dp71d were reported in PC12 cells [14, 21, 22]. It is proposed that in SGC7901 cells, by combinations of cell–environmental factors and the Dp71 amino acid sequence, enhanced Dp71d and Dp71f can both translocate into nucleus and form enhanced DAPC complex, which leads to nuclear structure and function alteration, and finally results in the same growth inhibition phenotypes of SGC-7901 cells.

As a key nuclear structure protein, lamin B1 plays an important role in the maintenance of cell proliferation and senescence [23, 24]. Previous research proved direct Dp71 association with lamin B1, emerin in PC12 cells [10]. Via evidence of immunoprecipitation, interaction between lamin B1 and Dp71 was also proved in GES-1 cells. Lamin B1 expression profile in our work is consistent with previous lamin B1 description in gastric cancer. Decreased LMNB1 is a poor prognosis marker in breast cancer [25,26]. Restoring lamin B1 expression in SGC7901 cells displayed the same proliferation inhibition traits as SGC7901-Dp71d and SGC7901-Dp71f cells. Significant lamin B1 RNA and protein fold increase was detected in SGC7901-Dp71d and SGC7901-Dp71f cells, which suggest Dp71 can regulate lamin B1 at RNA and protein level. Furthermore; we proved that knocking Dp71 expression down can attenuate the lamin B1 protein decrease in HBE and H9C2 cells under oxidative pressure (unpublished data). Our data suggests that Dp71 can also regulate the stability of lamin B1. Depletion of lamin B1 in mouse embryonic stem or HeLa cells led to mitotic defects, such as delayed pro-metaphase as well as abnormal mitotic spindle assembly, these mitotic defects may ultimately lead to impaired chromosome segregation [27-29], which is one of the most important causes initiating cancer. Increasing lamin B1 expression can cause the senescence of colorectal cancer cells [30]. Via decreasing the nuclear lamina protein lamin B1 from RNA and protein level, and further affecting its protein stability, decreased Dp71 expression caused by KLF4 and HNF3α will ultimately lead to the mitotic defects of normal gastric epithelium, which then leads to the initiation and progression of gastric cancer.

Interestingly, current research identified a cancer suppressive role of full-length dystrophin in myogenic cancer [31]. However, Dp71 was found unaltered in it, which means that defective expression of Dp71 is related only with certain types of cancers, such as gastric cancer, as shown by the our work. For the first time, we expand the biological role of Dp71 by proving its decreased expression in gastric cancer. Our data identified that the decreased Dp71 protein could be a prognosis biomarker for gastric cancer. Being one of the important nuclear proteins in spindle assembly, chromosome segregation, and post-mitotic nuclear assembly, it is highly possible that Dp71 protein complex identified in the nucleus plays an important tumor suppressive role in gastric cancer via changing expression of lamin B1 and other nuclear protein. Further studies are needed to clarify other nuclear proteins responsible for the tumor suppressive role of Dp71. Considering the ubiquitous expression of Dp71 in various tissues, further analysis of its expression in other cancers such as hepatic cancer, lung cancer, may further expand both the biology of Dp71, and cancer research as well.

## MATERIALS AND METHODS

### Human tissue specimens and ethic statement

34 pairs of patient gastric mucosa tissues samples from 2012 to 2013, 104 paraffin-embedded primary gastric carcinoma samples between 2009 and 2014 were collected, and analyzed in accordance with the guidelines of Xiangya Hospital. The study protocols were approved by the Research Ethics Committee of Central South University. None of these patients had received radiotherapy or chemotherapy prior to surgery. The histopathological type and stage of the gastric cancer were determined according to the criteria of the World Health Organization classification and the TNM stage set out by the Union for International Cancer Control.

### Cell lines, plasmids, antibodies and other reagents

The GES-1, SGC7901, BGC823 and AGS cell lines were purchased from Shanghai Type Culture Collection (Shanghai, China) and cultured as described in Supplementary Table S1. Anti-dystrophin antibody and Anti-lamin B1 antibody used were described in Supplementary Table 2. Dp71d and Dp71f plasmids were kind gifts from Dr Bulmaro Cisneros [9, 10]. Detailed information on lamin B1-pcDNA3.1, KLF4, and HNF3α were provided in supplementary materials and methods.

### Quantitative PCR

Real-time qPCR was performed as described previously [11]. The primers for Dp71, Lamin B1 and 18S can be found in Supplementary Table 3. RT-PCR data were normalized by measuring average cycle threshold (Ct) ratios between candidate genes and the control gene, 18S. The formula 2Ct (Candidate)/2Ct (Control) was used to calculate normalized ratios.

### Western blot analysis

The protein extraction and the whole experiment were performed as described previously [11]. The corresponding primary antibodies presented in Supplementary Table 2 were used in the experiment.

### Immunohistochemistry staining

Immunohistochemical staining of the 104 sections for Dp71 and 79 sections for lamin B1 (4 um thick) was performed as described in supplementary materials and methods. The detailed staining and scoring process and follow-up study can be found in Supplementary Materials and Methods.

### Immunoprecipitation

Total protein extracts in a final volume of 250 ml were incubated overnight at 4°C with 5 ug Rabbit anti-lamin B1 and 5ug Rabbit anti-Dp71 antibody, previously bound to protein G magnetic beads (Millipore). An irrelevant rabbit polyclonal antibody bound to protein G magnetic beads was performed as a negative control. The immune complexes were precipitated by placing the tube into the magnetic stand (Millipore) and washing 3 times with 500 μL of PBS containing 0.1% Tween 20. Precipitated proteins were separated by SDS-PAGE and analyzed by Western blotting with mouse anti-lamin B1 or mouse anti-Dp71 antibody.

### Cell viability CCK-8 assay

Cell viability was detected by Cell Counting Kit-8 (CCK-8, Beyond time, China), as previously described [12]. The assay was repeated 3 times.

### Bioinformatics analysis of the human Dp71 promoter region

Dp71 promoter region of human (GenBank accession number: NC_018934.2) was analyzed by the JASPAR (http://jaspar.genereg.net/) program and TFSEARCH (http://www.cbrc.jp/research/db/TFSEARCH.html) to predict transcription factor binding sites.

### Statistical analysis

A paired-samples t-test was used to compare the Dp71 mRNA levels in the tumor tissue samples and the adjacent non-tumor tissue samples. When the variables are quantitative, Pearson's correlation coefficients are used to analyze the relationship between Dp71 expression and various clinicopathological characteristics. But when the variables are categorical, association coefficients for contingency table are used to analyze the relationship. Overall survival curves are calculated with the Kaplan-Meier method and analyzed with the log-rank test. All statistical analyses were performed with SPSS software (version 17.0; SPSS Inc, Chicago, IL, USA).

## SUPPLEMENTARY MATERIALS FIGURES AND TABLES


