# Supplementary material for: Decreased Dp71 expression is associated with gastric adenocarcinoma prognosis
Source: Oncotarget. 2016 Jul 20;7(33):53702–11. doi: 10.18632/oncotarget.10724 (PMC5288215; doi:10.18632/oncotarget.10724)
Supplement: Supplementary file 1 [file oncotarget-07-53702-s001.pdf]

Decreased Dp71 expression is associated with gastric adenocarcinoma prognosis

SUPPLEMENTARY MATERIALS AND METHODS

Plasmids, siRNA and lipofectamine-mediated gene transfection

To generate lamin B1-pcDNA3.1 plasmids, a 1761 bp PCR fragment corresponding to human lamin B1 transcript (NM_005573) via amplification with the following primers: LMNB1-F:

GCCCTCTAGACTCGAGGCCACCATGGCGAC TGCGACCCCCGTGC;

LMNB1-R: GTAGTCACTTAAGCTTTTCATA ATTGCACAGCTTCTATTG. The PCR fragment were digested with *Xho*I and *Hin*dIII enzyme before subcloned in the correct reading frame into the expression vector pcDNA3.1 (Genechem ,Shanghai, China). All constructs were verified by restriction enzymes digestion analysis and sequencing. The construction of KLF4, HNF3α plasmids, HNF3α siRNA were describe in our previously published paper [1, 2]. The human KLF4 siRNA against human KLF4 (sc-35480) and its control were purchased from Santa Cruz Biotechnology. The application of the plasmids and siRNA were exactly the same as described in reference 1, 2.

Immunohistochemistry staining and follow-up

Immunohistochemical staining of the 104 sections for Dp71 and 79 sections for lamin B1 (4 um thick) was performed as follows: the formalin-fixed paraffin sections were deparaffinized with 100% xylene and rehydrated in descending percentage of ethanol series as described previously [3]. Heat-induced antigen retrieval was performed in 10 mM citrate buffer for 2 min at 100°C. Endogenous peroxidase activity and nonspecific antigen were blocked with peroxidase blocking reagent containing 3% hydrogen peroxide and serum, followed by incubation with rabbit anti-human Dp71 antibody (1:100, Abcam) , rabbit anti-human lamin B1 antibody(1:100, Bioworld) for overnight at 4°C.After washing, the sections were incubated with biotin-labeled goat anti-rabbit IgG antibody for 10 min at room temperature and subsequently conjugated with HRP (Maixin, China). Sections were visualized with DAB (3, 30-Diaminobenzidine) and counterstained with hematoxylin, mounted in neutral gum and analyzed using a bright-field microscope. The stained tissue sections were reviewed and scored independently by two pathologists blinded to the clinical parameters. The staining intensity was scored as 0 (negative), 1 (weak), 2 (medium) and 3(strong). The extent of the staining, defined as the percentage of positive staining areas in relation to the whole section area, was scored as “0” (5%, negative), “1” (5–25%, sporadic), “2” (25–50%, focal), or “3” (.50%, diffuse). The sum of the staining-intensity and staining-extent scores was used as the final staining score for Dp71. The total Dp71 immunostaining score ranged from 0 to 9. For statistical analysis, a final staining score of 0–4 and 5–6 of the gastric epithelium cells was considered to be low and high expression.

Follow-up data were obtained for all 104 patients. The follow-up period was defined as the interval between the date of operation and the date of the patient’s death or the last follow-up. The postoperative follow-up included clinical and laboratory examinations every 3 months for the first 2 years, every 6 months during the third to fifth years, and annually for an additional 5 years or until patient death, whichever occurred first. Overall survival, which was defined as the time from the operation to the patient’s death, or the last follow-up, was used as a measure of prognosis [3].

REFERENCES

1. Song L, Wei X, Zhang B, Luo X, Liu J, Feng Y, Xiao X. Role of Foxa1 in regulation of bcl2 expression during oxidative-s tress-induced apoptosis in A549 type II pneumocytes. Cell Stress Chaperones. 2009 Jul; 14:417-25.
2. Liu J, Yang T, Liu Y, Zhang H, Wang K, Liu M, Chen G, Xiao X. Krüppel-like factor 4 inhibits the expression of interleukin-1 beta in lipopolysaccharide-induced RAW264.7 macrophages.FEBS Lett. 2012 Mar 23; 586:834-40.
3. Ge J, Chen Z, Huang J, Chen ZK. Upregulation of Autophagy-Related Gene-5 (ATG-5) Is Associated with Chemoresistance in Human Gastric Cancer. PLoS One. 2014 Oct 17; 9:e110293.

SUPPLEMENTARY FIGURES AND TABLES

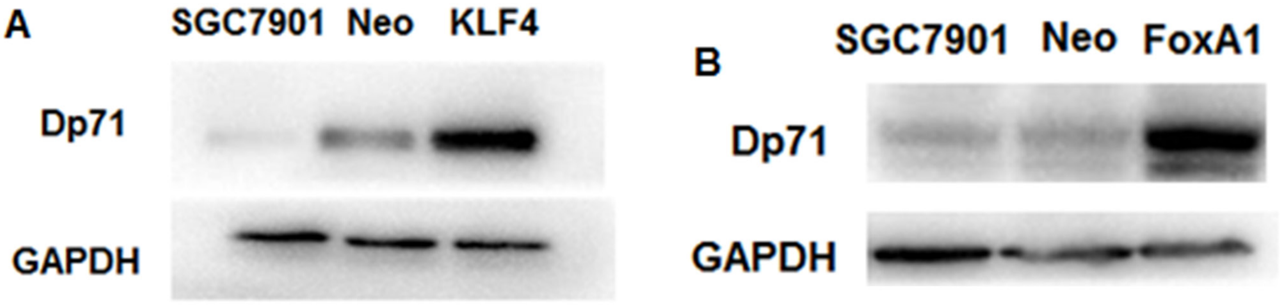

Supplementary Figure S1: Over-expression of KLF4 and HNF3α enhances Dp71 expression. As indicated in figure A and B of Figure S1, significant Dp71 protein increasing was displayed after transfection of KLF4 and HNF3α plasmids.

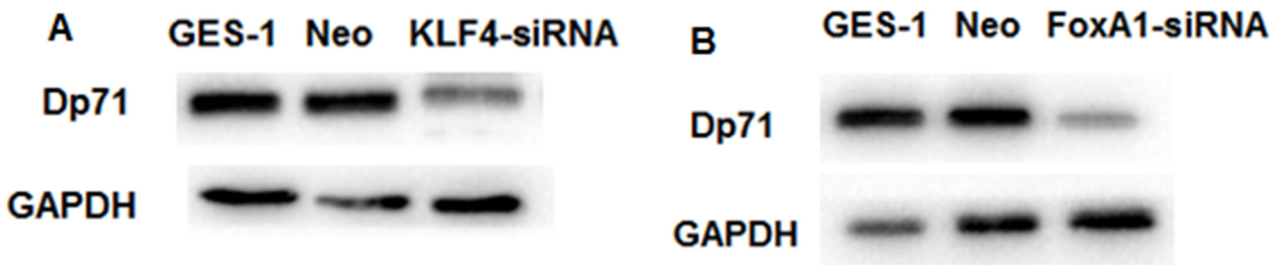

Supplementary Figure S2: KLF4 and HNF3α knock down suppress Dp71 expression. As indicated infigure A and B of Figure S2, significant Dp71 protein reduction was displayed after transfection of KLF4 and HNF3α siRNA.

Supplementary Table S1: Cell line subtypes and culture specifications. The GES-1, SGC7901, BGC823 and AGS cell lines were purchased from Shanghai Type Culture Collection (Shanghai, China). The detailed information of the cell lines is described as follows:

Cell lines	Cell Culture Specifications			
	Basal medium	Additives	Conditions	Passaging
**GES-1**	DMEM (Gibco)	10% FBS (Gibco)	5% CO2	0.25% trypsin(Gibco)
**SGC7901**	RPMI-1640(Gibco)	10% FBS	5% CO2	0.25% trypsin
**BGC823**	RPMI-1640	10% FBS	5% CO2	0.25% trypsin
**AGS**	RPMI-1640	10% FBS	5% CO2	0.25% trypsin

Supplementary Table S2: Antibody information

Antibody	Specificity	Nature	Usage in the paper And Dilution	Resource
**Dystrophin**	Dp427,Dp71d and Dp71f	Rabbit Polyclonal	WB:1/200 IP:1/100	Abcam(ab15277)
**Dystrophin**	Dp427,Dp71d and Dp71f	Mouse Monoclonal	WB:1/200	Abcam (ab7164)
**Lamin B1**	Lamin B1	Rabbit Polyclonal	WB:1/200 IP:1/100	Bioworld Technology, Inc (BS3547)
**Lamin B1**	Lamin B1	Mouse Monoclonal	WB:1/1000	Bioworld Technology, Inc (MB8006)

Supplementary Table S3: QRT-PCR Primers

Gene	Primer	Product length
Human Dp71 (NM_004015.2)	F:5’-TTGGCAGTCAAACTTCGGACTC-3’<br>R:5’-GTGTCCTCTCTCATTGGCTTTCCAG-3’	157bp
Human Lamin B1 (NM_005573.3)	F:5’TCCAGGAGAAGGAGGAGCTG3’<br>R:5’GGTCTCGTAGAGCGCCTTG3’	146bp
Human 18s (NM_022551)	F: 5’AA ATAGCCTTTG CCATCACTGCC 3’<br>R:5’GTTCAAGAAC CAGTCTGGGATC 3’	181bp
